# Metadata Correction: Mobile Phone Ownership Is Not a Serious Barrier to Participation in Studies: Descriptive Study

**DOI:** 10.2196/10403

**Published:** 2018-04-25

**Authors:** Emily J Harvey, Leslie F Rubin, Sabrina L Smiley, Yitong Zhou, Hoda Elmasry, Jennifer L Pearson

**Affiliations:** ^1^ Truth Initiative Schroeder Institute for Tobacco Research and Policy Studies Washington, DC United States; ^2^ Department of Psychology American University Washington, DC United States; ^3^ Department of Health, Behavior, and Society, Bloomberg School of Public Health Johns Hopkins University Baltimore, MD United States

In the metadata for “Mobile Phone Ownership Is Not a Serious Barrier to Participation in Studies: Descriptive Study” (JMIR Mhealth Uhealth 2018;6(2):e21), the degrees for Sabrina L Smiley were incorrectly listed as “MCHES, MPH, PhD”. The degrees should have been ordered “PhD, MPH, MCHES”.

The corrected article will appear in the online version of the paper on the JMIR website on April 25, 2018, together with the publication of this correction notice. Because this was made after submission to PubMed or Pubmed Central and other full-text repositories, the corrected article also has been re-submitted to those repositories.

